# X-Ray Dose Reduction in Abdominal Computed Tomography Using Advanced Iterative Reconstruction Algorithms

**DOI:** 10.1371/journal.pone.0092568

**Published:** 2014-03-24

**Authors:** Peigang Ning, Shaocheng Zhu, Dapeng Shi, Ying Guo, Minghua Sun

**Affiliations:** Department of Radiology, Henan Provincial People's Hospital, Zhengzhou, Henan, China; University of Washington School of Medicine, United States of America

## Abstract

**Objective:**

This work aims to explore the effects of adaptive statistical iterative reconstruction (ASiR) and model-based iterative reconstruction (MBIR) algorithms in reducing computed tomography (CT) radiation dosages in abdominal imaging.

**Methods:**

CT scans on a standard male phantom were performed at different tube currents. Images at the different tube currents were reconstructed with the filtered back-projection (FBP), 50% ASiR and MBIR algorithms and compared. The CT value, image noise and contrast-to-noise ratios (CNRs) of the reconstructed abdominal images were measured. Volumetric CT dose indexes (CTDIvol) were recorded.

**Results:**

At different tube currents, 50% ASiR and MBIR significantly reduced image noise and increased the CNR when compared with FBP. The minimal tube current values required by FBP, 50% ASiR, and MBIR to achieve acceptable image quality using this phantom were 200, 140, and 80 mA, respectively. At the identical image quality, 50% ASiR and MBIR reduced the radiation dose by 35.9% and 59.9% respectively when compared with FBP.

**Conclusions:**

Advanced iterative reconstruction techniques are able to reduce image noise and increase image CNRs. Compared with FBP, 50% ASiR and MBIR reduced radiation doses by 35.9% and 59.9%, respectively.

## Introduction

The development of computed tomography (CT) technology in recent years has revolutionized the field of radiology imaging and clinical diagnosis. CT scan has been extensively applied in clinical practice and is currently an indispensable tool for various clinical purposes. Its usage has been steadily increased annually. In United State alone, CT scan has been increased from estimated 1.3×10^7^ person-times scans in 1990 to 6.2×10^7^ in 2006 [1], [2]. As a consequence, concerns have been raised regarding the increased radiation exposure and risk of carcinogenesis [2], [3]. It is therefore critical to explore the methods or approaches to reduce the radiation exposure on both patients and medical professionals.

The International Commission on Radiation Protection has suggested that CT scanning practice should keep the radiation dose at the lowest achievable level to minimize the radiation exposure [4]. Namely the radiation doses should be reduced as much as possible on the premise of satisfying the clinical diagnostic requirements of imaging.

Abdomen is a common place for CT scans, from where a large range of multi-phase scan is routinely performed. In addition, multiple organs are located inside the abdomen, and each comes with diverse density, shape and components, they added up the complexity and requirement of high quality image for clinical evaluation and diagnosis. Previously strategies to achieve low-dose scans primarily include automatic tube current modulation, tube voltage reduction, pitch augmentation, and scan range control [5], [6]. These strategies however, all have limited effects on radiation dose reduction mostly due to the innate limitation of the traditional filtered back projection reconstruction (FBP) algorithm. Advanced iterative reconstruction algorithms have been well defined recently to solve the problem of image noise resulted from a reduced dose in CT with FBP algorithm, and possess the nature of producing high quality images at low dose radiation [7]–[10]. The purpose of this study was to evaluate the clinical values of the two advanced iterative reconstruction algorithms, namely the adaptive statistical iterative reconstruction (ASiR) and model-based iterative reconstruction (MBIR) algorithms, in reducing CT radiation dosages in abdominal imaging using a human abdominal phantom, and to determine the minimal required tube current for achieving acceptable image quality with different reconstruction algorithms.

## Materials and Methods

### Subject

**Figure 1:**
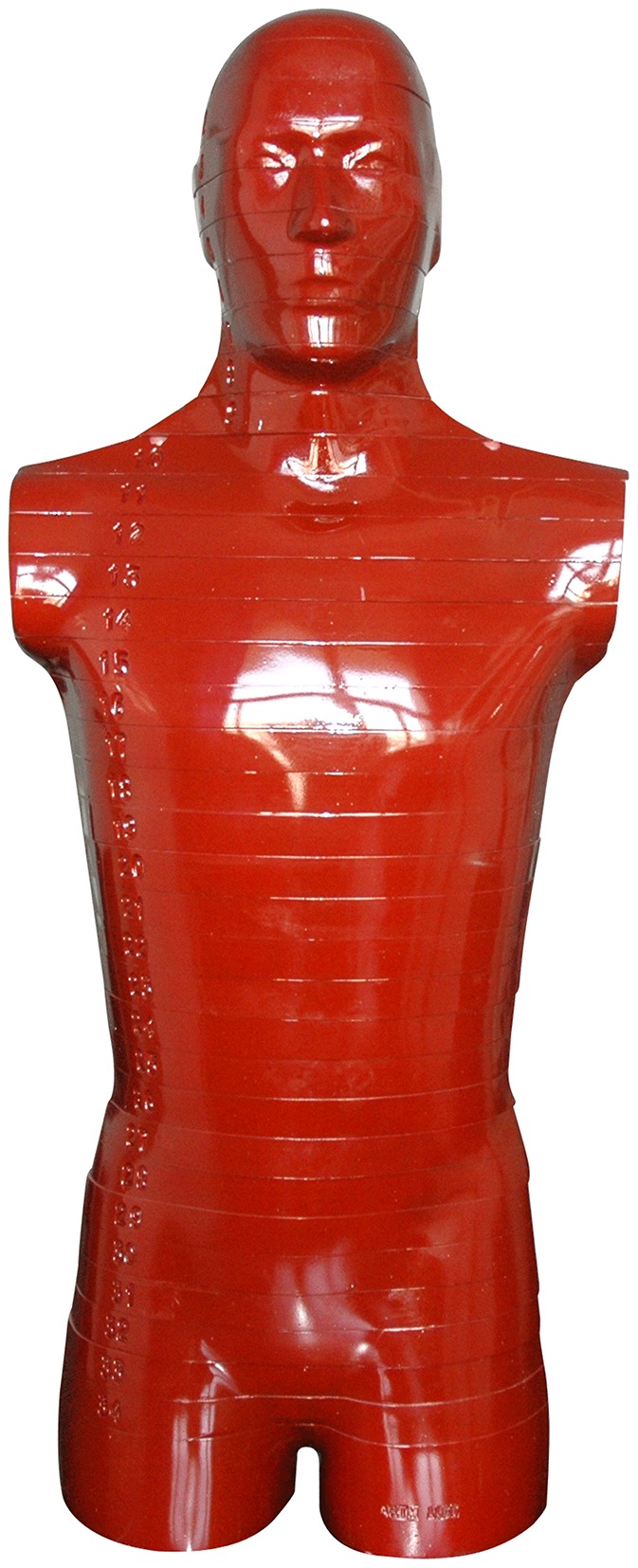
Biomedical RANDO standard male phantom.

A biomedical RANDO standard male phantom for radiotherapy with a height of 175 cm and a body weight of 73.5 kg (Beijing RGRMS, China) was used (Figure 1). The abdomen of the phantom is marked on specific area, with a circular hole for placing a radiation dose meter. Phantom was placed on the CT bed facing up, or supine position with a test tube filled with iodinated solution placed in the circular hole.

### Multislice CT and scanning scheme

The abdomen of the phantom was scanned repeatedly by a high-definition CT scanner (Discovery CT750 HD; GE Healthcare, USA) and then processed on an advanced image processing station (ADW4.5, GE Healthcare, USA). The tube currents were set at 400, 350, 300, 250, 200, 180, 160, 140, 120, 100, 80, 60, 50, 40, 30, 20, and 10 mA. Other parameters included a tube voltage of 120 kV, X-ray tube rotation time of 0.60 s, a pitch of 0.984, a slice thickness of 5 mm, an interlayer thickness of 5 mm, a matrix of 512×512, and a DFOV of 35 cm. The volumetric CT dose index (CTDIvol) at the different tube currents were recorded after each scan.

### Image processing and evaluation

The images obtained at the different tube currents were reconstructed using three different algorithms; FBP, 50% adaptive statistical iterative reconstruction (ASiR), and model-based iterative reconstruction (MBIR), with a reconstructed slice thickness of 0.625 mm. The reconstructed images were then transferred to the ADW4.5 workstation. An abdominal window with a level of 35 HU and a width of 400 HU was adopted for all the images. Five different bedding planes were selected from each image group for placingfive different regions of interest (ROI). The ROI was circular with an area of approximately 100 mm^2^. The copy and paste functions were used to propagate ROI into different sets of images to ensure the consistency and repeatability. Corresponding CT values, image noise (SD), and contrast-to-noise ratios (CNRs) were recorded. The CNR was calculated based on the following formula: CNR = 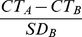; where *CT_A_* is the CT value of the iodinated solution inside a test tube on the measured bedding plane, *CT_B_* and *SD_B_* are the CT value and standard deviation of the background abdominal soft tissue. We also defined the noise decrease rate and CNR increase rate for the two iterative reconstruction (IR) algorithms using the values of FBP and reference standard: SD decrease rate _(IR)_ = (SD_FBP_-SD_IR_)/SD_FBP_×100%;CNR increase rate _(IR)_ = (CNR_IR_-CNR_FBP_)/CNR_FBP_×100%.

**Figure 2:**
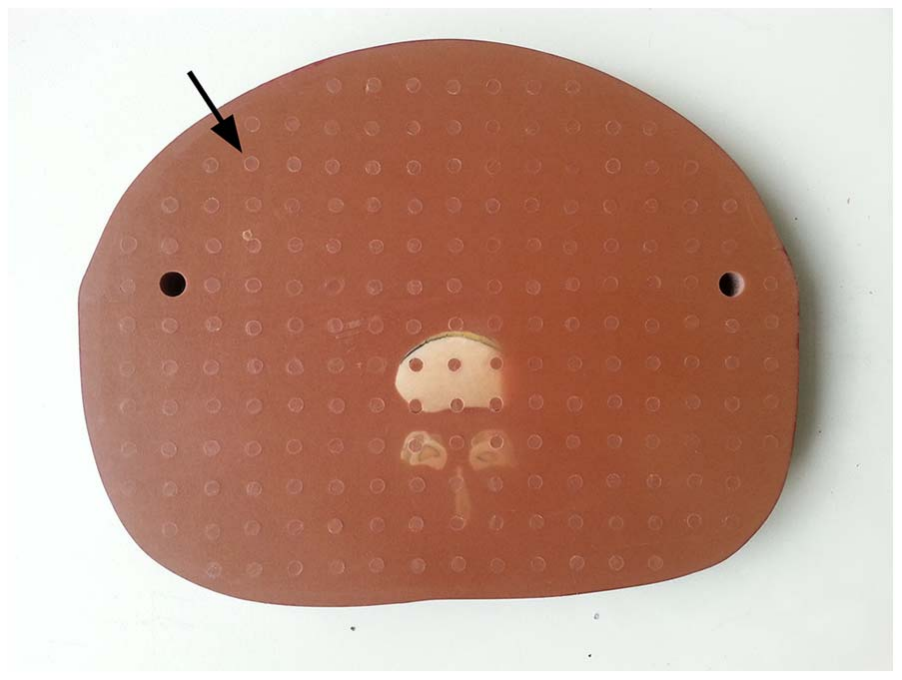
The cross section of the phantom. It has circular holes (arrows) to place the radiation dose meter. We evaluated the image quality about display of the circular hole (for placing a radiation dose meter) and the boundary of the image.

To increase the accuracy of the diagnosis, all of the images were evaluated by three senior radiologists who were blinded to the reconstruction algorithms. A 3-point scaling was employed to define the image quality, with score 1 being the best and score 3 the worst. Specifically, score 1 was defined as a clear image in which the circular hole (with the iodinated solution tube, Figure 2) was sharp and the boundary of the image was clear; score 2 was defined as the image had relatively high noise and circular hole was less sharp, but the boundary of the image was clear, which did not impair image observation; and score 3 was defined as image had relatively high noise with a fuzzy boundary and the circular hole was blurring, which impair the diagnostic observation. The evaluation results were analyzed to obtain the respective required minimal tube currents for diagnosis by the three reconstruction models. When there was disagreement, discussion and consultation were carried out for an agreement.

### Statistical analysis

Data are expressed as mean ±SD, all the qualitative and quantitative data obtained were used for comparing the FBP, ASiR, MBIR algorithms. The Difference between any two algorithms was determined by one-factor ANOVA by SPSS13.0 software. *P*<0.05 was considered statistically significant.

## Results

### Correlation between CTDIvol and the tube current

**Figure 3:**
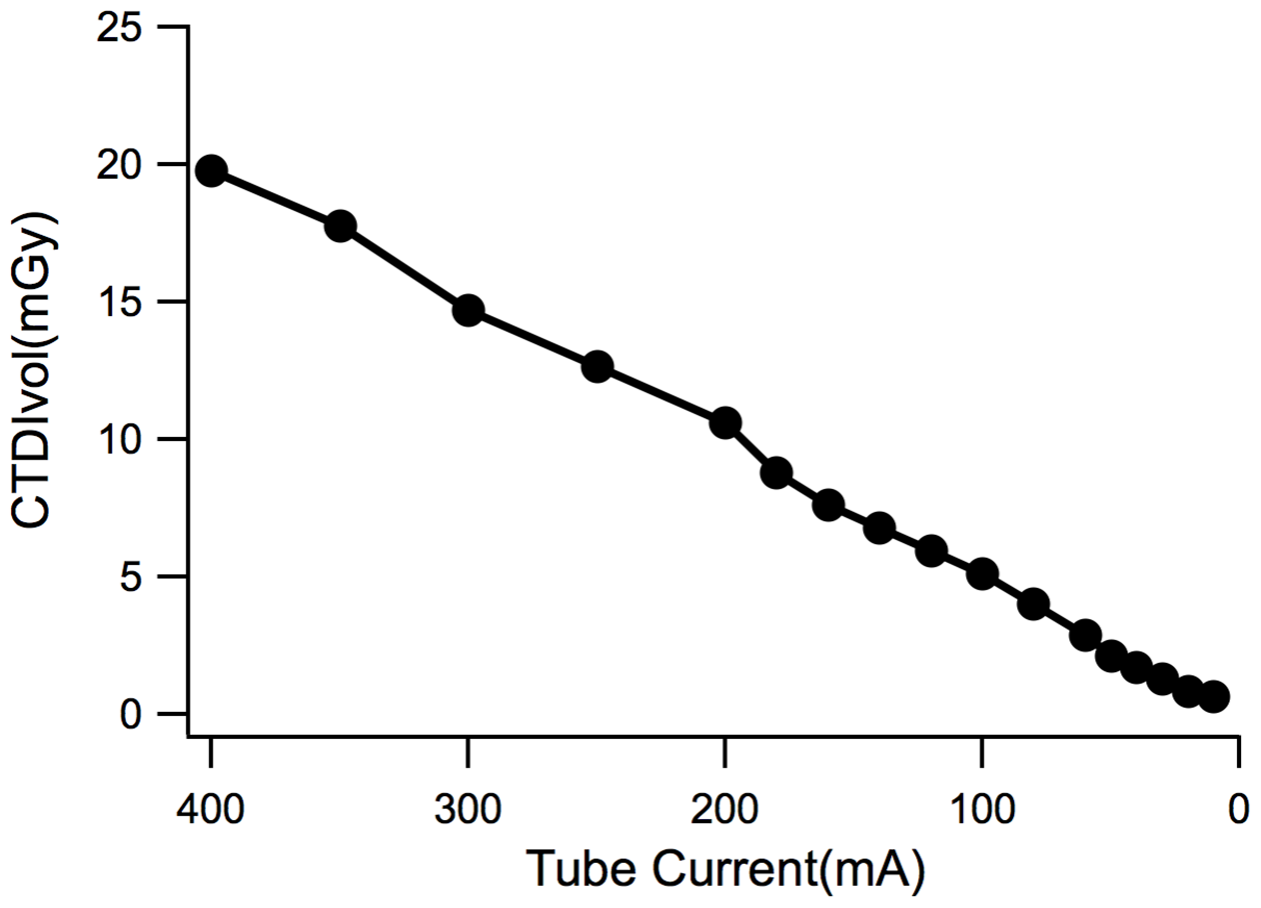
The schematic diagram of CTDIvol variations according to the mA value. CTDIvol decreases as the mA value decreases.

To test the correlation of CTDIvol and the tube current, we tested CTDIvol value in different tube currents to establish their relationship. The results showed that CTDIvol values changed proportionally to the changes of the tube current. CTDIvol value decreased gradually as the tube current decreased, the value reached the highest level of 20.6 mGy when tube current was set at 400 mA. The value dropped to the lowest level of 0.5 mGy when the tube current was set at 10 mA. Figure 3 showed the detailed trend of the correlation of the CTDIvol and the tube current.

### Image noise and CNRs analysis based on different reconstruction models

**Figure 4:**
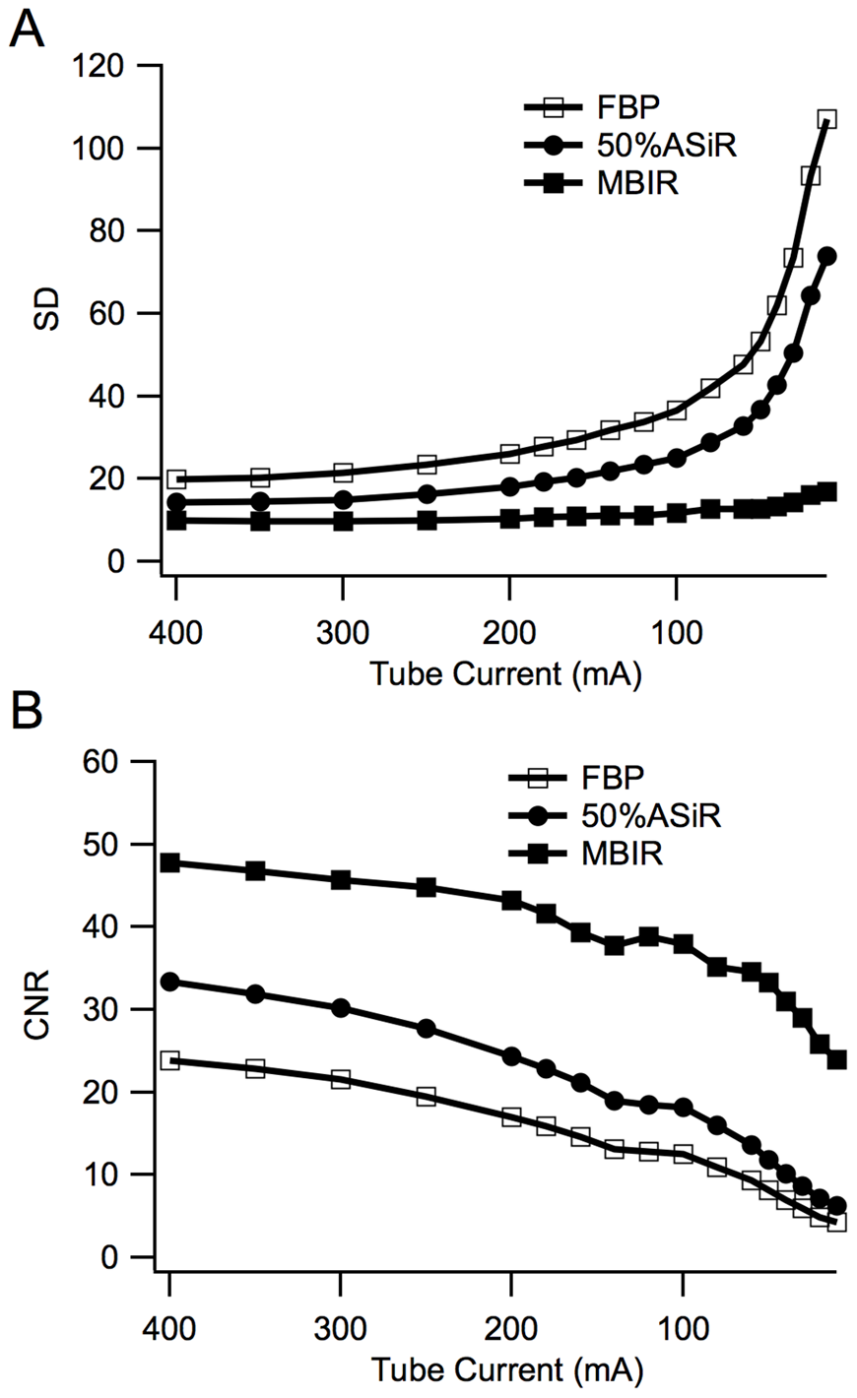
Image noise analysis based on the different reconstruction models. The schematic diagram of SD and CNR variations according to the mA values. All three reconstruction algorithms showed increased noise as the tube current decreased: FBP presented the most noticeable variations, followed by 50% ASiR and then by MBIR; the three models showed decreased CNRs as the tube current decreased, but at the same tube current, MBIR presented the highest CNR, which was followed by 50% ASiR and then by FBP.

All of the models presented in this work increased image noise with reduced CNRs as the tube current decreased (Figure 4). At the same tube current, the image noise and CNRs based on the different reconstruction models showed significant differences (*P*<0.05), but no differences were found in the CT values among the different reconstruction models (*P*>0.05).

### Noise and CNRs analysis under different tube currents

**Figure 5:**
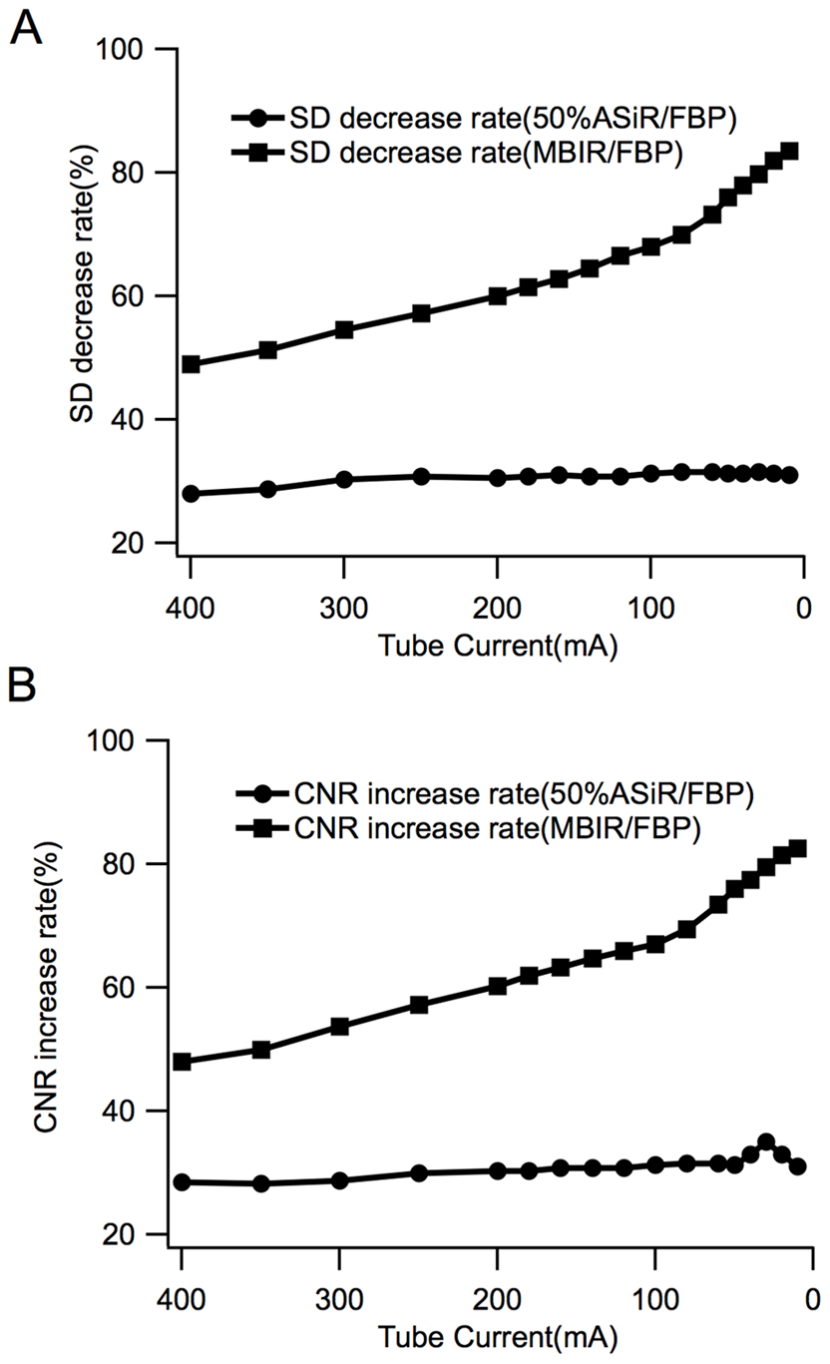
The schematic diagram of the SD decrease and CNR increase rates by 50% ASiR and MBIR (compared with those by FBP). As the mA value decreased, MBIR brought about gradually augmented variations in the two parameters, whereas 50% ASiR did not give rise to noticeable variations.

At the different tube currents (400−10 mA), the 50% ASiR algorithm decreased the image noise by 28%–32% when compared with the noise of FBP; and by MBIR model, the noise decreased by 45%–86%. We also compared the CNRs among three algorithms, the 50% ASiR increased the CNRs by 28%–32% when compared with FBP; and for MBIR, it increased by 46%–84% (Figure 5).

### Subjective assessments

According to the subjective assessments, the minimal required diagnostic radiation doses by FBP, 50%ASiR, and MBIR algorithms were 200 mA, 140 mA, and 80 mA, respectively. At the identical image quality, the doses that required by the 50%ASiR and MBIR decreased by 35.9% and 59.9% when compared with that by FBP algorithm.

## Discussion

CT radiation dose can be affected by multiple factors, such as tube voltage, tube current, time of exposure, scan range, pitch, X-ray energy, scattered X-ray content, the size of the collimator, structure of the front ray filter, and geometric size of the scanner [5], [6]. When the scanning conditions are kept consistent, the radiation dose is increased as the tube current increasing. In the present study, we calculated and analyzed the CTDIvol values for phantom scans at different tube currents, the results showed that radiation doses were positively correlated with tube currents. Therefore, decreasing the tube current can be an effective strategy to reduce the radiation doses.

The principle of the FBP algorithm is based on the assumption that projection data have no image noise, whereas the noise is an innate property of projection data; therefore, the traditional FBP algorithm is subject to noise generation and artifacts. Considering the fact that image noise increases as the tube current decreasing, proper radiation dose is prerequisite to ensure good quality of images [11]. In contrast to FBP algorithm, advanced iterative reconstruction algorithms require much less projection data and enable images to be reconstructed with incomplete data even at lower radiation doses. They reconstruct images in high quality image based on scarce sampled data and achieve satisfactory clinical images at even greatly reduced radiation doses [12], [13].

By establishing the noise models of the scanned objects, ASiR employs the iterative calculation method and effectively reduce image noise while maintaining image resolution [7], [14], [15]. The application of ASiR in abdominal CT scan at a low voltage allows for greater reduction of image noise, better image quality, and decreases the radiation doses from 17.5 mSv to 5.1 mSv [9]. Abdominal CT using ASiR algorithm decreases the required radiation dose by 25.1% and meanwhile greatly reduces image noise when compared with FBP method [16]. In addition, ASiR has been shown to reduce image noise and enhances the assessment capacity of a coronary artery stent in vitro [17]. In the current study, we noted that 50% ASiR reduced the reconstructed image noise from 28% to 32% and increased the CNR from 28% to 39% at the same tube current when compared with FBP algorithms. In addition, 50% ASiR reduced the radiation dose by 35.9% when identical image quality was maintained.

MBIR is an advanced iterative reconstruction algorithms, it uses five models including the three-dimensional optical models and noise model during iterative reconstruction to remove statistical noise and optical fuzzy effect from raw data [18], [19]. Since MBIR does not blend with filtered back projection components, it is therefore more complicated and precise than ASiR. MBIR significantly reduces image noise and streak artifacts, enhances spatial resolution, and further reduces radiation dose without sacrificing the quality of image [12], [20]. In the current study, MBIR reduced the radiation dose from 5.54 mSv to 1.13 mSv and image noise by 80% compared with ASiR algorithm [18]. A previous study shows that when compared with FBP, MBIR achieved satisfactory image quality with a minimal tube current of only 50 mA and reduced the radiation dose by 75% [21]. Our results also showed that MBIR reduced image noise from 45% to 86%, enhanced the CNR from 46% to 84% at the same tube current, and reduced the radiation dose by 59.9% at the identical image quality when compared with FBP algorithm.

Furthermore, our results also indicated that FBP image noise was increased as the tube current decreasing. We noted that 50% ASiR decreased the noise by 30% and this noise decrease rate did not depend on the tube current values when it was compared with FBP algorithm. In contrast to 50% ASiR, the noise decreasing rate by MBIR increased gradually as the tube current value decreased, the noise reduction rate was 54% when the conventional radiation dose for an abdominal examination (300 mA) was selected, while when the dose was adjusted to 80 mA, the noise reduction rate increased to 69%.

The subjective assessments in this study revealed that the respective minimal tube current values for the interest of diagnosis by FBP, 50% ASiR and MBIR were 200 mA, 140 mA, and 80 mA, respectively. Therefore, MBIR-based reconstructed images had the lowest noise level, but the highest CNR and hence, the lowest radiation dose among all the three algorithms evaluated, indicating that MBIR is a much superior algorithm in reducing the radiation dose over ASiR and FBP.

However, our study has some limitations. First, as we used phantom as a model, which only has a simple structure such as soft tissue and bone, rather than human abdominal organs and fat, therefore, the results remain to be confirmed in clinical practice. Second, because MBIR reconstruction model requires large amount of calculation, longer time are expected during the generation of image at X-ray optical system and data collection. The reconstruction of MBIR was about 1 h per case, equaled to 0.09 frame/s. The reconstruction speed of FBP was 15 frames/s, and that of ASiR was 10 frames/s [9], [18]. For this reason, this technique has not been extensively applied in clinical practice. Nonetheless, we believe the reconstruction speed of MBIR will be continuously improved with the development of novel computer technology [18], [22].

To summarize, both 50% ASiR and MBIR algorithms can significantly decrease the image noise compared with FBP, and MBIR has a better effect in lowing the radiation doses. At the identical image quality, 50% ASiR and MBIR reduce the radiation dose by 36% and 60% over the FBP algorithm.
